# Differential Hepatic Expression of miRNA in Response to Aflatoxin B1 Challenge in Domestic and Wild Turkeys

**DOI:** 10.3390/toxins16110453

**Published:** 2024-10-22

**Authors:** Kade Jorud, Kristelle M. Mendoza, Thomas Kono, Roger A. Coulombe, Kent M. Reed

**Affiliations:** 1 College of Veterinary Medicine, University of Minnesota, St Paul, MN 55108, USA; 2 Department of Veterinary and Biomedical Sciences, University of Minnesota, St. Paul, MN 55108, USA; 3 Minnesota Supercomputing Institute, University of Minnesota, St Paul, MN 55108, USA; 4 Department of Animal, Dairy and Veterinary Sciences, Utah State University, Logan, UT 84322, USA; roger@usu.edu

**Keywords:** aflatoxin B1, domesticated turkey, wild turkey, liver, RNA-seq, microRNA

## Abstract

Aflatoxin B1 (AFB_1_) is a major foodborne mycotoxin that poses a significant economic risk to poultry due to a greater degree of susceptibility compared to other agricultural species. Domesticated turkeys (*Meleagris gallopavo*) are especially sensitive to AFB_1_; however, wild turkeys (*M. g. silvestris*) are more resistant. A lack of functional isoforms of hepatic glutathione S-transferases (GSTs), an enzyme that plays a role in the detoxification of aflatoxin, is suspected as the reason for the increased sensitivity. Previous studies comparing the gene expression of domesticated and wild turkeys exposed to AFB_1_ identified hepatic genes responding differentially to AFB_1_, but could not fully explain the difference in response. The current study examined differences in the expression of microRNAs (miRNAs) in the livers of wild and domesticated turkeys fed dietary AFB_1_ (320 μg/kg in feed). Short-read RNA sequencing and expression analysis examined both domesticated and wild turkeys exposed to AFB_1_ compared to controls. A total of 25 miRNAs was identified as being significantly differentially expressed (DEM) in pairwise comparisons. The majority of these have mammalian orthologs with known dysregulation in liver disease. The largest number of DEMs occurred between controls, suggesting an underlying difference in liver potential. Sequences of the DEMs were used to identify potential miRNA binding sites in target genes, resulting in an average of 4302 predicted target sites per DEM. These DEMs and gene targets provide hypotheses for future investigations into the role of miRNAs in AFB_1_ resistance.

## 1. Introduction

Aflatoxin (AFB_1_) is a mycotoxin of significant economic importance to the poultry industry. Previous studies have identified differential susceptibility to AFB_1_ between domesticated (*Meleagris gallopavo*) and wild turkeys (*M. g. silvestris*). In humans and in most animals, resistance is determined primarily by the expression of protective hepatic AFB_1_-detoxifying glutathione S-transferases (GSTs). Livers of domesticated turkeys lack AFB_1_-detoxifying activity, whereas wild turkeys express functional AFB_1_-protective GSTs [1,2,3]. Recombinant GSTAs cloned from domesticated and wild turkeys are comparable in function (in vitro), unlike the hepatic forms (in vivo) from which they were amplified, implying that these enzymes are downregulated, silenced, or otherwise modified by one or more possibly epigenetic mechanisms [4]. In a series of studies, we have investigated the comparative molecular responses in the hepatic and splenic transcriptomes directly affected by dietary challenge to AFB_1_ in turkeys and in developing turkey embryos. Pathways significantly dysregulated by AFB_1_ in the liver included cancer, apoptosis, cell cycle, and lipid regulation, reflecting molecular mechanisms of inflammation, proliferation, and liver damage in aflatoxicosis [5,6,7]. In the spleen, AFB_1_ suppressed innate immune transcripts, especially from antimicrobial genes indicative of either increased cytotoxic potential or activation-induced cell death during aflatoxicosis [8]. In an in ovo exposure model, controlled AFB_1_ exposure of developing embryos similarly revealed differential responses in wild vs. domesticated birds [9].

The unique susceptibility of domesticated turkeys to the effects of AFB_1_ compared to wild turkeys is likely due to a lack of functional hepatic GSTs. Extended studies of juvenile birds aimed at contrasting the gene expression response of susceptible (domesticated) to more resistant wild birds by characterizing transcriptional changes induced by AFB₁ in the liver, spleen, and cecal tonsil [10,11,12]. RNA sequencing found statistically significant differences in gene expression in AFB₁-treated birds compared to controls. Characterization of the differentially expressed genes found dysregulation in response to AFB_1_ with significant association of Phase I and Phase II genes and genes associated with cellular regulation, modulation of apoptosis, and inflammatory responses. The expression of *GSTA3* was significantly higher in AFB₁-treated birds versus controls for both genetic groups. Interestingly, wild birds had higher *GSTA3* expression compared to domesticated birds, even when fed the control diet. Results of this study supported the hypothesis that the greater resistance of wild turkeys to AFB_1_ is related to a higher constitutive expression of *GSTA3* coupled with other differences in functional gene expression and that these are likely caused by downregulation, gene silencing, and/or other mechanisms [13].

The non-coding RNAs, microRNAs (miRNAs), are a class of small (18–25 nucleotides) regulatory RNAs that play an important role in controlling the abundance of transcripts in the vertebrate transcriptome [14]. By recognizing target sites predominantly in the 3′UTRs of mRNAs, these molecules bind to destabilize mRNAs, resulting in mRNA cleavage or posttranscriptional repression of gene expression [15]. Gene silencing mediated by miRNAs plays an important role in animal development and disease [16] and in the animal response to environmental stressors [17,18,19,20].

Tissue-specific expression of miRNAs is common in vertebrates [21], and relatively little is known about their expression in the turkey. Characterization and computational prediction of miRNA is the first step in identifying miRNA:mRNA target interactions. Previous work by our group examined the role of miRNAs expressed in skeletal muscle satellite cells [22], where more than 350 miRNAs were identified through small RNA sequencing. Differential expression of several miRNAs was found in response to thermal stress, suggesting an important role for miRNAs in response to heat stress (altered cellular proliferation and differentiation) with notable differences in birds selected for their modern commercial growth traits. Studies in humans and model species such as mice and rats have shown that the expression of miRNAs and miRNA:mRNA interactions are specifically altered in almost all liver diseases [23]. This study was designed to characterize the expression of miRNAs in turkey liver in the context of AFB_1_ exposure. Here, we performed miRNA-seq on the same animals used in the challenge studies described above [10,11,12] and contrasted miRNA expression in both wild and domesticated birds. We hypothesized that the expression of miRNAs would be significantly altered by AFB_1_ treatment and would vary between domesticated and non-selected wild birds.

## 2. Results

### 2.1. Small RNA Sequencing

Results of small RNA sequencing are summarized in Table 1. The number of raw sequence reads per library ranged from 5.4 M to 17.7 M, with the total number of reads being slightly higher in the EW control and EW-AFB_1_ treatment groups with an average of 10.47 M reads per library. Read quality was consistently high, with an average mean Q score of 37.

### 2.2. Identification and Expression of Conserved and Novel miRNAs

**Figure 1:**
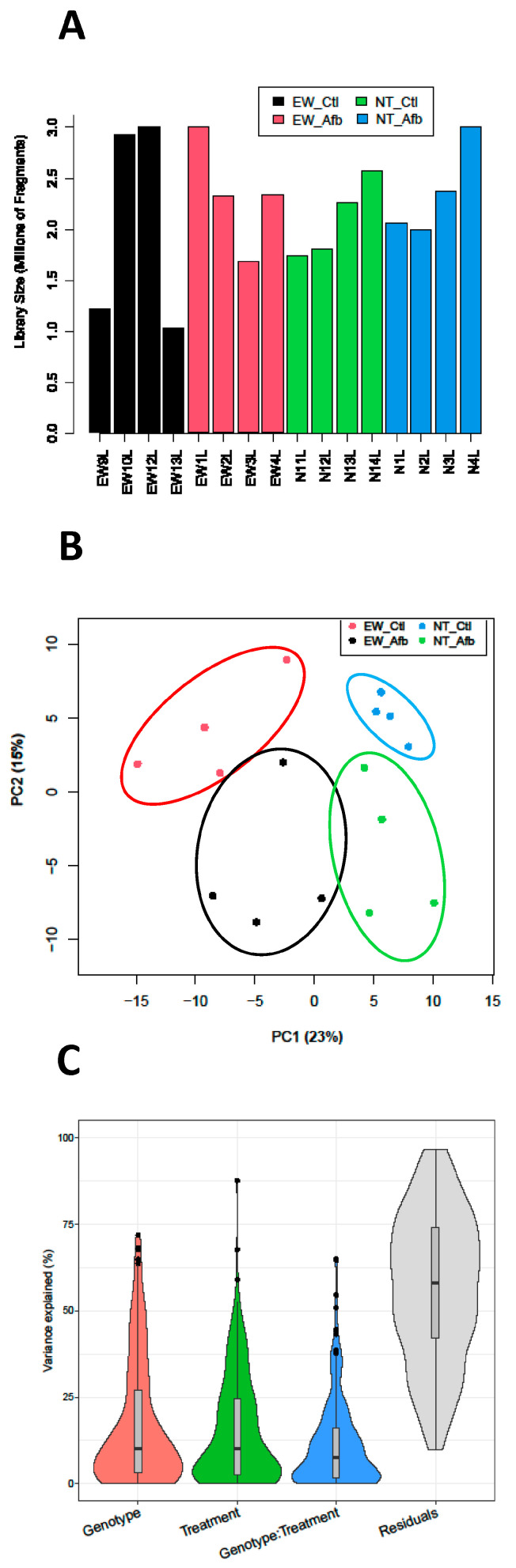
(**A**). Distribution of sequencing reads (Millions of fragments) in sequencing libraries of treatment groups (Control and AFB_1_). (**B**). Principal component analysis (PCA) plots of normalized read counts. Sample-to-sample distances (within- and between-treatments) are illustrated for each treatment sample on the first two principal components and plotted according to treatment. (**C**). Distribution of sample variance by treatment factor: Genotype (Line, Eastern Wild (EW) and Nicholas (NT) turkeys), AFB_1_ treatment, interaction, and residual.

Clean reads obtained from all sequencing libraries were used for miRNA prediction using miRDeep2. Although the total number of reads mapping to the miRNA precursors varied across libraries, the averages per treatment group were similar, ranging from 2.04 M to 2.36 M reads (Figure 1A). The performance of miRDeep2 in the detection of known miRNAs (those identified based on sequence comparison of their miRNA precursors with previously reported turkey miRNAs and the *G. gallus* in the miRBase dataset) and novel miRNAs is presented in Table S1. A total of 529 miRNAs (52 known and 477 novel) was detected (Supplementary Materials File S1) across all libraries. Combined, this represented 451 unique miRNAs (41 known and 410 novel) with duplicates removed. The expression of the putative novel miRNAs was lower than that of the known miRNAs.

Novel miRNAs were considered as high confidence when the putative mature and star miRNA sequence were detected in at least two independent samples, having the exact same 5′- and 3′-ends with no mismatches. Based on cutoff values for confidence (miRDeep score > 1.0, significant RNAfold *p*-value, and mature reads > 10), 322 of the 477 detected novel miRNAs (67.5%) were considered high confidence.

**Figure 2:**
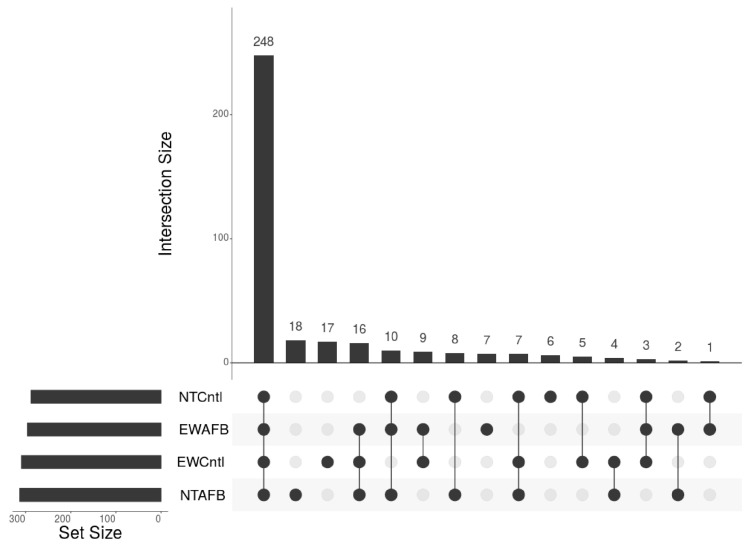
UpSet plot of expressed miRNAs in turkey liver. For inclusion, miRNAs must first have at least three assigned reads in at least two libraries and, secondly, a treatment group with an average number of reads > 2.0. The horizontal bars on the left (Set size) indicate the number of miRNAs expressed in each treatment. Individual points in the matrix represent miRNAs expressed in each treatment, and the lines between points represent shared expression. The vertical bars above indicate the number of miRNAs specific to or common to different treatments.

The distribution of the expressed miRNAs is summarized in Figure 2. Of the 529 predicted miRNAs, the expression of 168 (31.7%) fell below our analysis cutoff. Of the remaining 361 miRNAs, 248 (46.8%) were expressed in all treatment groups, 32 were expressed in 3 groups, 29 in two groups, and 48 were uniquely expressed in single treatments (Figure 2). The principal component (PCA) plot shown in Figure 1B visualizes variation in expression among treatment groups. Here, treatment groups clustered distinctly with separation by genotype along the first principal component (PCA1) and separation by treatment along PCA2. The variance partition plot (Figure 1C) shows a similar contribution of genotype and AFB_1_ treatment to the overall experimental variance.

### 2.3. Identification of Differentially Expressed miRNAs (DEMs)

A counts matrix of 238 miRNAs was created from normalization by library size for analysis with EdgeR (Table S2). A total of 25 miRNAs with significant differential expression (DEMs) was identified in the treatment group comparisons (Table 2, Figure S1). In the control birds, 15 DEMs occurred between the NT domestic and EW wild birds, with 8 being upregulated and 7 downregulated in the NT domestic birds relative to the EW wild birds. Four of these, miR-N210, miR-138 (2 isoforms) and miR-190a had Log_2_ fold-change (Log_2_FC) > 1.0 and five, miR-29b, miR-27b, miR-N424, miR-1768 and miR-N224, had Log_2_FC < −1.0. Five DEMs occurred in the comparison of control vs. AFB_1_-treated NT domestic birds (Table 2). These included miR-30d, miR-125b-5p, miR-99a, miR-221 and miR-N60. With the exception of miR-30d, these showed increased expression with AFB_1_ treatment. Only a single DEM (miR-N60) occurred in comparison of control vs. AFB_1_-treated EW wild birds. This miRNA, identified as novel in our previous study [22], had seed match similarity to chicken miR-3529 but significant BLAST similarity to gga-miR-7-1. This DEM (likely a 7-1 ortholog) was similarly regulated with higher expression in the AFB_1_ treatment groups in both lines (Log_2_FC = −1.5247). Finally, comparison of the AFB_1_-treated animals found six DEMs (Table 2). With the exception of miR-130b, these showed higher expression in the EW birds than NT domestic.

### 2.4. MicroRNA Target Predictions

The mature consensus sequences of the 25 DEMs identified in the pairwise comparisons were used to query the transcript sequences in the turkey genome for potential target sites. Target predictions identified 61,527 potential interactions (alignment score > 150) involving 8933 genes. The number of target sites per miRNA ranged from 251 (miRNA-99a) to 8780 (two isoforms of miR-138), with an average of 2641. Gene transcripts containing predicted sites with the highest alignment score (≥170) are highlighted in Table S3. The highest target scores were observed for mga-miR-211 (SLC6A12, solute carrier family 6 member 12), mga-miR-1768 (LOC100542432, histamine N-methyltransferase-like), mga-miR-221 (GABRD, gamma-aminobutyric acid type A receptor delta subunit), mga-miR-181b (COMP, cartilage oligomeric matrix protein), and mga-miR-30d (LOC100541720, Fanconi-associated nuclease 1).

For the 15 DEMs of the control comparisons (NT vs. EW), 41,302 gene target interactions were predicted with MiRanda. Of these, 749 (occurring in 394 genes) had alignment scores ≥ 170. Interestingly, one DEM (mga-miR-27b) had predicted target sites in two alpha-class GSTs (GSTA3 and GSTA4), although with somewhat lower alignment scores (160 and 152, respectively). Predicted target-binding sites for the five DEMs in the NT domestic bird comparison (control vs. AFB_1_-treated) totaled 6353, with 138 (occurring in 83 genes) having alignment scores ≥ 170. Of the 138 high-score targets, 26 were predicted for miR-N60, which was also found in the control vs. AFB_1_-treated comparison in EW wild birds. One DEM (mga-miR-221) in the NT domestic birds also had a target site in GSTA3 (alignment score = 150). Finally, in the six DEMs of the AFB_1_-treated bird comparison (NT vs. EW), 13,872 targets were predicted, with 254 (occurring in 185 genes) having alignment scores ≥ 170.

GO analysis of the high score (≥170) gene targets did not indicate significant gene enrichment for any of the comparison sets. However, combined GO analysis of the top-scoring targets (Table S4) found significant (FDR = 2.52 × 10^−2^) enrichment (36.73×) for the GO molecular function category. This combined GO analysis also found histone methyltransferase binding and several GO biological function categories, including oxalate transport (27.55×, FDR = 3.49 × 10^−2^), aspartate transmembrane transport (13.36×, FDR = 4.20× 10^−2^), C4-dicarboxylate transport (13.12×, FDR = 1.60 × 10^−2^), and dicarboxylic acid transport (10.49×, FDR = 5.43 × 10^−5^). Functional annotation clustering of the 3000 genes (2900 DAVID IDs) with the top target MiRanda alignment scores in DAVID found one KEGG annotation cluster (enrichment score: 1.81×) that included the pathways mgp01040:Biosynthesis of unsaturated fatty acids (12 genes, P = 1.26 × 10^−2^), mgp01212:Fatty acid metabolism (18 genes, P = 1.45 × 10^−2^), and mgp00062:Fatty acid elongation (11 genes, P = 2.04 × 10^−2^).

## 3. Discussion

Non-coding RNAs help regulate diverse biological functions, including cell proliferation, differentiation, cell death, organ development, and physiology. Downregulation of miRNAs typically increases the translation of target genes, whereas upregulation has a negative effect. Importantly, as negative regulators of gene expression, miRNAs can function as tumor suppressors or oncogenes. In humans, it is estimated that 70% of all described miRNAs are expressed in the liver, with some, such as miR-122, comprising > 70% of all hepatic miRNAs [24]. In the past 20 years, the number of miRNA studies has grown exponentially, with several studies demonstrating altered the expression of miRNAs in numerous acute and chronic liver diseases [23]. Due to their stability in body fluids, miRNAs are also useful as biomarkers for liver dysfunction.

The most likely mechanism for the extreme sensitivity of domesticated turkeys to AFB_1_ is dysregulation/dysfunction in hepatic GSTs, rendering them unable to detoxify AFB_1_ [1,2,3,4,25]. The liver is the principal site of AFB_1_ metabolism. In the turkey, AFB_1_ is first bioactivated by high-efficiency cytochrome P450s (1A5 and 3A37) to the reactive and electrophilic exo-AFB1-epoxide (AFBO) [26,27,28,29]. Subsequent binding of AFBO to DNA and other macromolecules results in immunotoxicity, mutations, and aflatoxicosis [5,30]. Aflatoxicosis in the turkey is characterized by an enlarged and pale liver resulting from increased vacuolation of AFB_1_ -exposed hepatocytes with accumulation of high levels of lipids (reviewed in Monson et al. [31]. As in human liver diseases, dysregulation of miRNAs in the turkey was hypothesized in response to AFB_1_ exposure.

### 3.1. Differential Effects of AFB_1_ Challenge

Many vertebrate genes are miRNA targets [32], and numerous studies have now demonstrated associations between miRNA expression and human liver disease. Many miRNAs and their targets are highly conserved [33], and the majority of DEMs identified in this study have mammalian orthologs associated with human liver disease. For example, a comparison of NT domestic birds fed AFB_1_ found four such DEMs (mga-miR-99a, mga-miR-221, mga-miR-125b-5p, and mga-miR-30d) compared to controls. In humans, the expression of miR-99a correlates with the inhibition of hepatocellular carcinoma (HCC) by inducing the G(1) phase cell cycle arrest [34]. In addition, Zhang et al. [35] found that a central component of the RNA-induced silencing complex (Argonaute-2, *AGO2*), was translationally repressed by miR-99a in HCC. Similarly, studies have found miR-221 to be a biomarker of chronic liver injury [36], and it is dysregulated in multiple cancers [37]. This miRNA is upregulated in liver fibrosis, and overexpression contributes to liver tumorigenesis [38]. In the case of the third DEM (miR-125b-5p), Hua et al. [39] demonstrated this miRNA as a tumor suppressor in HCC through inhibition of the antioxidant thioredoxin reductase 1 (*TXNRD1*). Yang et al. [40] also found miR-125b-5p to regulate hepatocyte proliferation during liver regeneration by targeting ankyrin repeat and the BTB/POZ domain containing protein 1 (*ABTB1*). Target prediction in the turkey also found *ABTB1* as a predicted target of mga-miR-125b-5p (alignment score = 154, Table S3). Orthologs of the single miRNA with lower expression in NT domestic birds fed AFB_1_ (mga-miR-30d) also play a role in HCC. By targeting the G protein (*Galphai2*, GNAI2), miRNA-30d promotes tumor invasion and metastasis [41].

Following AFB_1_ challenge, a single miRNA (mga-miR-130b) was expressed to a higher degree in the NT domestic than in EW birds. As with other miRNAs, miR-130b is associated with human HCC and is significantly dysregulated in tumors [42]. In HCC cells, miR-130b expression is inversely correlated with peroxisome proliferator-activated receptor gamma (*PPAR*-γ) [43]. Targeting and inhibition of PPAR-γ, a regulator of adipocyte differentiation, promotes HCC aggressiveness (cell migration and invasion). Although *PPAR*-γ was not predicted as a target of turkey mga-miR-130b, *PPAR*-α transcripts were (alignment score = 152). miR-130b is also a stimulator of hepatic very-low-density lipoprotein assembly and secretion [44].

Furthermore, two miRNAs (mga-miR-29c, mga-miR-204) had lower expression in NT domestic birds compared to EW birds. As a member of the miR-29 family, miR29c is also associated with HCC by directly targeting oncogenic sirtuin 1 (*SIRT1*, [45]). Inhibition by miR-204 of the key enzyme in the carnitine-dependent transport pathway (cpt1a) in mouse hepatocytes promotes non-alcoholic fatty liver disease [46]. *CPT1A* was predicted as a potential target for eight of the turkey DEMs identified in this study. Finally, an ortholog of the third DEM (mga-miR-1388) regulates the expression of antiviral genes via tumor necrosis factor receptor-associated factor 3 in fish [47].

A single DEM (mga-miRNA-N60) with significantly higher expression with AFB_1_ treatment was shared between the NT and EW control vs. AFB_1_ comparisons. As mentioned above, this miRNA is an ortholog of miR-7-1. In mammals, miR-7-1 is regulated by PPAR-α, and has been shown to control cell growth, proliferation, invasion, metastasis, metabolism, and inflammation [48]. miR-7 also promotes hepatocellular lipid accumulation [49] and has tumor-suppressive effects in hepatocarcinogenesis through the suppression of the oncogene cyclin E1 (*CCNE1*, [50]).

### 3.2. Genetic Background and Hepatic miRNA Expression

The majority of miRNAs with elevated expressions in the control group of NT domestic birds also have mammalian orthologs with demonstrated roles in liver disease. Higher expression of this group of miRNAs (Log_2_FC = 0.48 to 1.61, Table 2) in the NT domestic controls would likely result in reduced expression in their target genes and is the result of the genetic background and history of commercial selection in these birds as compared to their wild counterparts. For example, the DEM ortholog (miR-128) suppresses *CYP2C9*, the most abundant CYP2C subfamily enzyme in the human liver [51]. Huang et al. [52] found miR-128-3p to regulate phosphoinositide-3-kinase regulatory subunit 1 (*PIK3R1*) and suppress the proliferation of HCC. Two isoforms of mga-miR-138 were upregulated in control turkeys. This miRNA induces cell cycle arrest in HCC by targeting cyclin D3 [53]. Both miR-190a and miR-429 are involved in HCC tumor-related biological processes, including proliferation, apoptosis, metastasis, and drug resistance [54,55,56,57]. In contrast, miR-181b has been shown in mice to target early growth response 1 (*EGR1*), and inhibition of miR-181b-5p reduces glycogenesis in hepatocytes through the AKT/GSK pathway [58]. This miRNA activates the PTEN/Akt pathway in stellate cells during the initiation and progression of liver fibrosis [59]. *EGR1* was identified as a potential target for two other DEMs identified in this study (mga-miR-N224 and mga-miR-N424). The last DEM (mga-miR-1559) does not have a known mammalian ortholog but is found in both the chicken and zebra finch genomes (miRbase, [60]).

In control turkeys, the miRNAs with lower expression in NT domestic compared to EW also have human orthologs associated with HCC promotion. Lower expression of this group of miRNAs in the NT domestic (Log_2_FC = −0.79 to −2.66) would likely result in increased expression in their target genes. For example, miR-23b is significantly downregulated in primary HCC [61], miR-24 promotes cell growth and is a prognostic indicator for multiple cancers [62], and miR-27b-3p inhibits HCC by targeting TGF-Beta Activated Kinase 1 (*MAP3K7*) Binding Protein 3 (*TAB3*, [63]). *TAB3* was predicted as a potential target for seven of the turkey DEMs identified in this study, including mga-miR27b. When inhibited, miR-27b-3p, miR-24, and miR-23b also mediate hepatic lipid accumulation and hyperlipidemia [64,65,66]. One of the most abundantly expressed miRNAs in the human liver is the miR-29 family [67]. The expression of miR-29b reduces liver fibrosis [68,69]. The remaining DEM in this group (mga-miR-1768) occurs in both chicken and zebra finch but does not have a known mammalian ortholog.

This study identified miRNAs expressed in turkey liver, characterized differential expression in response to AFB_1_, and predicted miRNA:mRNA interactions. Our prior studies supported the contention that the expression of *GSTA3* coupled with other differences in functional gene expression underlie the differences in AFB_1_ susceptibility between domesticated and wild turkeys. With the exception of mga-miR-27b, we did not identify evidence for robust targeting of *GSTA3* transcripts by the DEMs, as the mga-miR-27b binding sites had lower-quality alignment scores. However, the suite of DEMs identified in the present study provides insight into the biological response to AFB_1_ and how commercial selection for the modern domesticated turkey has altered this response.

Although not mechanistically tested, our analyses identified several DEMs and a number of genes potentially targeted by miRNAs following AFB_1_ exposure. For example, in addition to *GSTA3*, potential DEM target sites for 10 other genes of the *Glutathione Conjugation* super pathway (*CHAC1*, *CNDP2*, *ESD*, *GCLM*, *GGCT*, *GSS*, *GSTA4*, *GSTK1*, *GSTZ1*, and *MGST2*) are included in our predicted DEM target-binding sites. Although target sites for miRNAs are amongst the most highly conserved motifs within mRNA 3′UTRs [15], the functionality of individual miRNAs may be different in this species compared to mammals. In any event, further validation of miRNA/mRNA interactions is needed. This resource provides new hypotheses for future research, which are supported by the link of the turkey DEMs identified in this study to orthologs implicated in human liver disease.

## 4. Materials and Methods

This study used RNA samples extracted from our previous study of two turkey subspecies in the context of AFB_1_ treatment [10]. These subspecies had previously demonstrated varying AFB_1_-detoxifying GST activity. Briefly, hatchling birds were acclimated for two weeks on an ad libitum standard grow-up soy-based diet. Males from each line (n = 8 for Eastern wild (EW) and n = 10 for Nicholas Turkey (NT, domestic) were then assigned to one of two treatment groups (Control of AFB_1_) and the AFB_1_ groups (EW-AFB_1_ and NT-AFB_1_) subjected to a short-term AFB_1_-treatment protocol [1]. The diet of challenge birds was amended beginning on day 15 with 320 μg/kg (320 ppb) AFB_1_ (Sigma-Aldrich, Inc., St. Louis, MO, USA) for 14 days. The AFB1 concentration used in the challenge study was chosen with the aim of observing sub-clinical molecular pathologies while avoiding significant clinical hepatotoxic effects [70]. Control birds continued on the standard diet with AFB_1_ levels below detection limits (<10 μg/kg, 10 ppb) based on testing of feed via HPLC [10]. Birds were euthanized, and livers were removed and infused with RNAlater (Thermo Fisher Scientific, Waltham, MA, USA) for subsequent RNA isolation.

### 4.1. RNA Isolation and Sequencing

Total RNA was isolated using RNAzol RT extraction, DNase-treated (Turbo DNA-free Kit, Thermo Fisher Scientific Wilmington, DE, USA), and stored at −80 °C until use. Initial RNA concentration and quality were assessed using spectrophotometry (Nanodrop 1000, Thermo Fisher Scientific Wilmington, DE, USA), and samples were submitted for QC and library preparation and sequencing at the University of Minnesota Genomics Center. Each sample was quantified by RiboGreen Assay (Thermo Fisher Scientific Wilmington, DE, USA) on the 2100 Bioanalyzer (Agilent Technologies, Santa Clara, CA, USA) to confirm RNA integrity. Indexed libraries (n = 16, 4 per treatment group) were constructed with the TruSeq smRNA library preparation kit (Illumina, Inc., San Diego, CA, USA) and size selected. Libraries were multiplexed and sequenced on the HiSeq2500 platform (Illumina, Inc.) to produce 50-bp single-end reads (data accessioned as part of NCBI SRA BioProject 342653).

### 4.2. Illumina Sequencing Data Handling

Low-quality bases and adaptor contamination were removed from the Illumina sequencing reads and with FastQC 0.11.9 (https://www.bioinformatics.babraham.ac.uk/projects/fastqc/, accessed 20 October 2024) and FastQC reports combined with MultiQC 1.13 [71]. Sequencing adaptors were removed with cutadapt 4.2 [72]. Reads shorter than 15 nucleotides after trimming were removed and cleaned of ribosomal sequences with BBDuk 39.01 (https://sourceforge.net/projects/bbmap/, accessed 20 October 2024) using reference sequences (large and small ribosomal subunit) retrieved from SILVA release 132 [73]. Reads with an exact match (min 15 nt) to one of the reference sequences were removed. Only reads trimmed of adaptors and depleted of ribosomal sequences were used downstream for analysis.

### 4.3. miRNA Prediction

For the prediction of novel miRNAs against the turkey genome, cleaned reads from all libraries were combined into a single file. The turkey genome assembly (GCA_943295565.1) was prepared for mapping with Bowtie 1.3.1 [74]. Novel miRNAs were predicted using miRDeep2 0.1.2 [75], and miRNA sequences with miRDeep2 scores > 0 were retained. Characterized mature miRNAs from turkey [22] and from chicken (*Gallus gallus*) (downloaded from miRBase release 22.1) were used as previously known miRNAs. Sequence similarity of the novel miRNAs to those predicted previously in Reed et al. [22] was further confirmed via sequence alignments in Sequencher (v 5.1, Gene Codes, Corp, Ann Arbor, MI, USA).

### 4.4. miRNA Expression Profiling

Reads from each library were separately mapped to the turkey genome with Bowtie 1.3.1 [74] using the options “-n 0 -e 80 -l 15 -m 5 --best --strata” to recreate the parameters used for miRNA discovery. SAM to BAM file conversion was conducted with Samtools 1.14 [76], and parallel processing of alignment files was performed with GNU parallel version 20210822 [77]. miRNA regions identified by miRDeep2 were converted to SAF files for expression quantification with ‘featureCounts’ version 2.0.3 [78]. Only reads with a minimum mapping quality of 10 were counted. Expression values were normalized by library size (total number of reads mapping to miRNA precursors) and multiplied by a factor of 1 × 10^6^ (corresponding to counts per million mapped miRNA reads, CPM). Differentially expressed miRNAs (DEMs) were determined with the ‘edgeR’ package [79] in the R statistical computing environment version 4.2.2 [80] using the counts matrix from featureCounts. MicroRNAs with low expression (at least three assigned reads in at least two libraries) were filtered. Differential expression analyses were carried out with the quasi-likelihood F test in edgeR [81]. Global miRNA expression was assessed with principal components analysis using the prcomp() function in R. Variance partitioning analyses were conducted with the ‘variancePartition’ package [82] in R to estimate the contributions of genotype, AFB_1_ treatment, and an interaction of genotype and AFB_1_ treatment to variance in miRNA expression.

### 4.5. miRNA Target Prediction

Potential miRNA target genes were predicted in MiRanda 2.0 by aligning the miRNA sequences against RNA transcripts in the annotated UMD5.1 genome build (NCBI annotation 104) with position-weighted scoring, alignment score > 150 and |Energy-Kcal/Mol| > 7.0. Enrichment tests for target genes were performed using the PANTHER Overrepresentation Test [GO Consortium release 20150430 [83,84]; http://geneontology.org/, accessed 20 October 2024]. GO analysis utilized the chicken (*G. gallus*) reference gene set. Functional annotation of target genes was performed with DAVID [85,86].

## Figures and Tables

**Table 1:** Summary of RNA-seq data used for miRNA discovery and expression analysis. For each library, the total number of sequence reads, percent GC content, and mean quality (Q score) are given.

Group	Replicate	Total Reads	% GC	Mean Read Quality
EW-Control	EW9L	14,151,896	55	36.8
	EW10L	17,696,678	55	36.9
	EW12L	14,450,425	54	37.0
	EW13L	5,439,559	55	36.9
EW-AFB_1_	EW1L	14,406,168	54	36.9
	EW2L	12,879,470	54	36.9
	EW3L	8,418,981	55	37.0
	EW4L	6,979,042	53	37.1
NT-Control	N11L	8,598,754	54	37.0
	N12L	6,605,326	54	37.0
	N13L	10,318,300	54	37.0
	N14L	12,049,804	54	37.0
NT-AFB_1_	N1L	6,796,414	54	37.0
	N2L	9,351,794	54	37.0
	N3L	9,381,403	54	36.9
	N4L	10,029,675	54	37.0
Mean		10,472,105.6	54.2	37.00

**Table 2:** Differentially expressed miRNAs (DEMs) in turkey liver.

Comparison	Turkey miRNA	Similar Seed Match	Log_2_FC	FDR
NT vs. EW (Control)	mga-miR-N210	NA	1.6067	0.0302
	mga-miR-138	gga-miR-138-5p	1.1568	0.0106
	mga-miR-138	gga-miR-138-5p	1.1254	0.0106
	mga-miR-190a	gga-miR-190a-5p	1.0744	0.0123
	mga-miR-429	mga-miR-429	0.7412	0.0353
	mga-miR-181b	gga-miR-181a-5p	0.7256	0.0353
	mga-miR-1559	gga-miR-1559-5p	0.7221	0.0353
	mga-miR-128	gga-miR-128-3p	0.4804	0.0353
	mga-miR-24	gga-miR-24-3p	−0.7883	0.0123
	mga-miR-23b	gga-miR-23b-3p	−0.9695	0.0106
	mga-miR-29b	gga-miR-29a-3p	−1.0786	0.0152
	mga-miR-27b	gga-miR-27b-3p	−1.1868	0.0106
	mga-miR-N424	NA	−1.5619	0.0152
	mga-miR-1768	gga-miR-1768	−1.5732	0.0406
	mga-miR-N224	NA	−2.6583	0.0106
NT (Control vs. AFB_1_)	mga-miR-30d	gga-miR-30d	0.4608	0.0175
	mga-miR-125b-5p	gga-miR-125b-5p	−0.4150	0.0407
	mga-miR-99a	gga-miR-99a-5p	−0.6275	0.0250
	mga-miR-221	gga-miR-222a	−1.2598	0.0415
	mga-miR-N60	gga-miR-3529	−1.4936	0.0002
EW (Control vs. AFB_1_)	mga-miR-N60	gga-miR-3529	−1.5247	0.0298
NT vs. EW (AFB_1_)	mga-miR-130b	gga-miR-130b-3p	0.5476	0.0482
	mga-miR-204	gga-miR-204	−0.7917	0.0482
	mga-miR-N195	NA	−0.7982	0.0482
	mga-miR-29c	gga-miR-29a-3p	−0.8797	0.0482
	mga-miR-N63	mga-miR-N63	−1.5288	0.0482
	mga-miR-1388a	gga-miR-1388a-5p	−1.8226	0.0278

## Data Availability

The original data presented in the study are openly available in NCBI’s Sequence Read Archive (SRA), accessioned as part of SRA BioProject PRJNA342653.

## Supplementary Materials

The following supporting information can be downloaded at: https://www.mdpi.com/article/10.3390/toxins16110453/s1, Figure S1: MA plot showing the relationship between average count (logCPM) and fold-change (log2FC) across the miRNAs; File S1: miRDeep summary results; Table S1: Summary of miRDeep2 performance; Table S2: Normalized read counts; Table S3: Predicted target gene of differentially expressed turkey miRNAs (DEMs); Table S4: PANTHER Overrepresentation Test for target genes of turkey miRNAs.
