# Supplementary figures and images for: Differential Hepatic Expression of miRNA in Response to Aflatoxin B1 Challenge in Domestic and Wild Turkeys
Source: Toxins (Basel). 2024 Oct 22;16(11):453. doi: 10.3390/toxins16110453 (PMC11598555; doi:10.3390/toxins16110453)

**A****EW\_Ctl VS NT\_Ctl**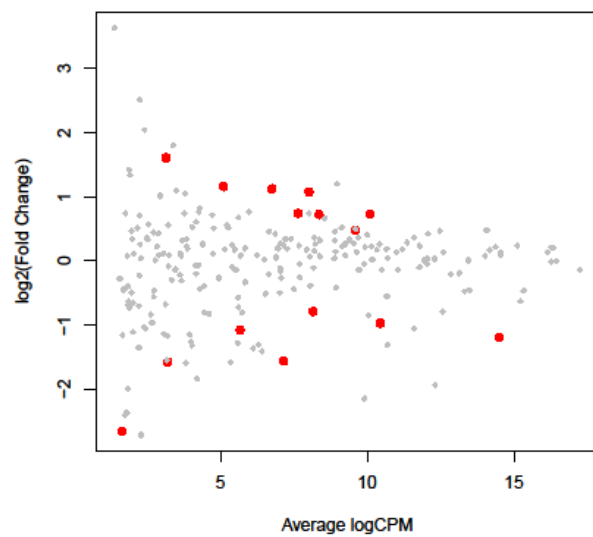**B****EW\_Afb VS EW\_Ctl**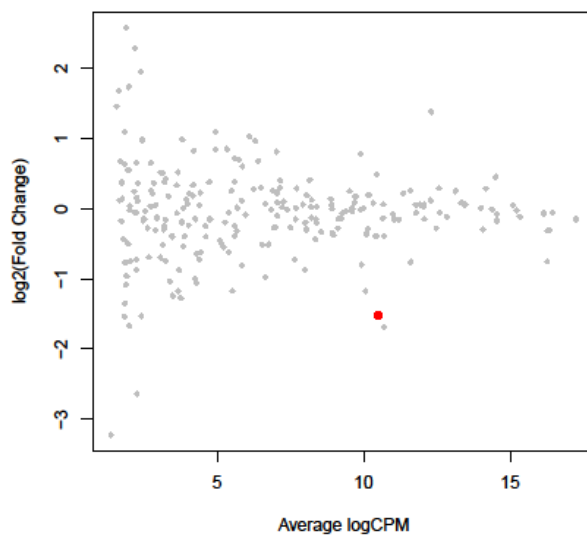**C****NT\_Afb VS NT\_Ctl**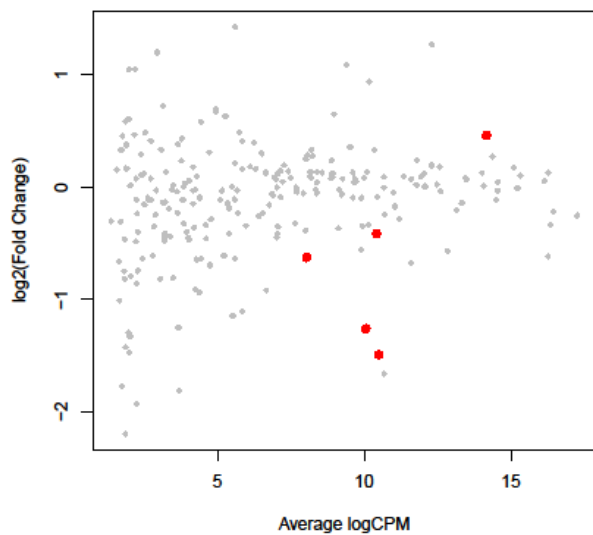**D****EW\_Afb VS NT\_Afb**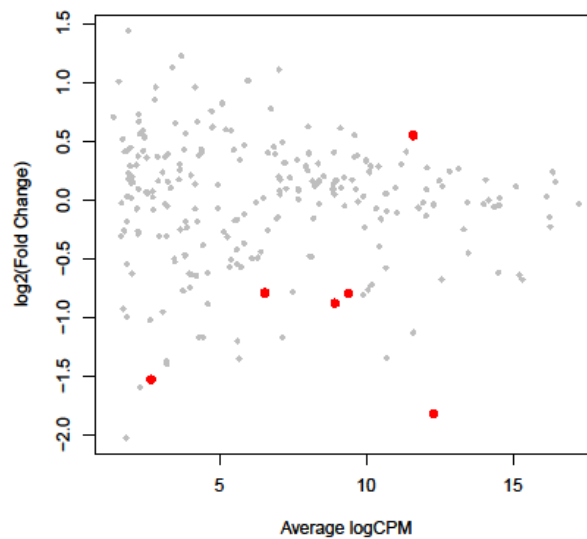

Supplement: Supplementary file 1 [file toxins-16-00453-s001.zip › Figure S1.pdf]
